# Identification of a novel cellobiose 2-epimerase from *Acidobacteriota bacterium* and its application for *in-situ* milk catalysis

**DOI:** 10.3389/fmicb.2025.1575725

**Published:** 2025-04-04

**Authors:** Qiuqian Zeng, Xiaomei Lyu

**Affiliations:** ^1^School of Food Science and Technology, Jiangnan University, Wuxi, China; ^2^State Key Laboratory of Food Science and Resources, Jiangnan University, Wuxi, China; ^3^Yixing Institute of Food and Biotechnology, Wuxi, China

**Keywords:** cellobiose 2-epimerase, acidophilic enzyme, milk processing, low-lactose milk, epilactose, prebiotics

## Abstract

**Introduction:**

Cellobiose 2-epimerase (CE) catalyzes the interconversion of glucosyl and mannosyl groups at the reducing end of *β*-1,4-linked disaccharides. This enzyme is pivotal for converting lactose into prebiotics like epilactose, offering a potential solution for lactose-intolerant-friendly dairy products. However, current CEs are hindered by pH and thermal instability in milk processing, as their neutral-to-alkaline pH optima clash with milk’s mildly acidic conditions (pH 6.5–6.7), and their poor thermolability requires costly post-processing enzyme removal.

**Methods:**

We identified a novel CE from the acidophilic *Acidobacteriota bacterium* (Acba-CE) and characterized its properties. Its enzymatic activity was assessed under varying pH and temperature conditions, including milk-processing environments.

**Results:**

Acba-CE exhibits an acidic pH optimum (6.0), retaining 95% activity at milk pH (6.5). Notably, it undergoes rapid thermal inactivation at pasteurization temperatures, enabling complete enzyme deactivation during standard pasteurization without additional steps. In milk systems, Acba-CE achieves 28.5% lactose-to-epilactose conversion at refrigeration temperatures (10°C), demonstrating strong cold adaptability.

**Discussion:**

To our knowledge, this is the first reported CE from the *Acidobacteriota* phylum, combining acidophilic activity with low-temperature adaptability. Acba-CE represents a breakthrough for *in situ* dairy modification, eliminating key bottlenecks in milk processing and enabling next-generation functional milk production.

## 1 Introduction

Cellobiose 2-epimerase (CE, EC 5.1.3.11) is a class of enzymes that plays a pivotal role in carbohydrate metabolism. Initially identified in *Ruminococcus albus* (Kim et al., 2012; Ito et al., 2007), CE belongs to the N-acetyl-glucosamine 2-epimerase (AGE) superfamily. These enzymes are widely distributed in nature, particularly among microorganisms, and demonstrate notable catalytic diversity with significant potential for industrial applications. The primary function of CE is to catalyze the interconversion of glucosyl and mannosyl groups at the reducing end of disaccharides linked by *β*-1,4-glycosidic bonds (Fujiwara et al., 2014). Common substrates catalyzed by CE include cellobiose, lactose, and 4-O-β-D-mannosyl-D-glucose. When lactose serves as the substrate, the epimerization activity of CE catalyzes its conversion into epilactose. As a derivative of lactose, epilactose is a non-digestible disaccharide with prebiotic properties, exhibiting various health-promoting bioactivities (Xiong et al., 2024), such as regulating intestinal flora (Watanabe et al., 2008) and promoting calcium absorption (Nishimukai et al., 2008; Suzuki et al., 2010). In recent years, research on the bioconversion of this sugar has garnered widespread attention, with CE playing a crucial role in this process (Pang et al., 2025).

Beyond its application in the industrial production of epilactose, CE is now being considered for use in the processing of milk (Eat et al., 2024; Krewinkel et al., 2014; Rentschler et al., 2015). Milk is abundant in lactose, but some individuals lack the enzyme lactase, which is primarily produced by cells in the small intestine, to digest it. When these individuals consume milk containing lactose, due to insufficient secretion or reduced activity of lactase, a significant amount of lactose remains undigested and accumulates in the intestine, potentially leading to adverse symptoms such as bloating, abdominal pain, diarrhea, nausea, and vomiting (Szilagyi and Ishayek, 2018; Angima et al., 2024).

**Figure 1:**
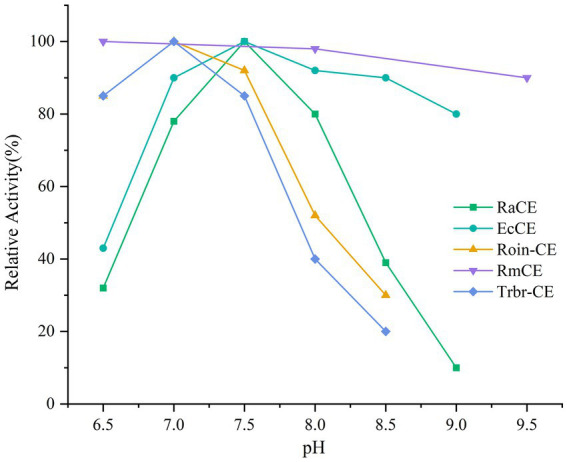
Relative activity of CEs from different sources at various pH levels. RaCE, *Ruminococcus albus*; EcCE, *Eubacterium cellulosomes*; Roin-CE, *Roseburia intestinalis*; RmCE, *Rhodothermus marinus*; Trbr-CE, *Treponema brennaborense*.

The addition of CE to milk could enable *in situ* conversion of lactose into prebiotic epilactose. However, this strategy faces a critical challenge: milk’s mildly acidic pH (6.5–6.7) drastically reduces the activity of conventional CEs, which typically exhibit neutral-to-alkaline pH optima. Consequently, these enzymes experience varying degrees of activity loss in acidic environments. Table 1 summarizes the enzymatic properties of the main CE enzymes currently used for epilactose production. Figure 1 illustrates the relative activity of CE enzymes from different sources at various pH levels. Although the CE enzyme derived from *Rhodothermus marinus* (Ojima et al., 2014) is suitable for acidic environments, its optimal temperature is too high, making it difficult to inactivate during processing at high temperatures and unsuitable for use in milk processing environments.

**Table 1:** Enzymatic properties of cellobiose 2-epimerase (CE) enzymes from different sources.

Organism	Optimum pH	Optimum Temperature (°C)	K_m_	k_cat_	k_cat_/K_m_	References
*Ruminococcus albus*	7.5	30	33	52.1	1.6	Kim et al. (2012) and Ito et al. (2007)
*Pedobacter heparinus*	6.3	35	24.5	5.43	0.222	Krewinkel et al. (2014)
*Eubacterium cellulosomes*	8	35	72	32.5	0.451	Taguchi et al. (2008)
*Roseburia intestinalis*	7	45	429.9	117	0.27	Chen et al. (2020)
*Rhodothermus marinus*	6.3	80	28.8	111	3.85	Ojima et al. (2014)
*Bacteroides fragilis*	7.5	45	6.56	79.5	12.1	Senoura et al. (2014)
*Thermoanaerobacterium saccharolyticum*	7	60	124.7	30.9	0.248	Chen et al. (2015)
*Treponema brennaborense*	7	45	57.8	135.1	2.347	Chen et al. (2021)

To identify enzymes that maintain high activity in slightly acidic environments, the exploration of novel CEs sourced from microorganisms adapted to acidic conditions is considered. This study focuses on the CE derived from *Acidobacteriota bacterium* (Acba-CE), examining its role in catalyzing the conversion of lactose to epilactose. As a recently classified bacterial phylum, *Acidobacteriota bacterium* is categorized as acidophiles, capable of surviving and reproducing in acidic environments (Ludwig et al., 2006). This unique physiological characteristic enables enzymes derived from *Acidobacteriota bacterium* to exhibit a degree of acidophilia, thus better adapting to and being applicable in slightly acidic industrial production environments.

In this research, our objective was to gain insights into the catalytic properties and application potential of Acba-CE, particularly its advantages in acidic environments. We conducted extensive expression and purification of this enzyme in *Escherichia coli* host, conducted in-depth investigations into its enzymatic properties and activity, and achieved efficient conversion of lactose to epilactose within a short period. The ultimate goal is to facilitate its widespread application in fields such as food, pharmaceuticals, and health supplements.

## 2 Materials and methods

### 2.1 Chemicals, materials, and strains

Isopropyl *β*-D-1-thiogalactopyranoside (IPTG), Kanamycin (Kana), Piperazine-N,N′-bis(2-ethanesulfonic acid) (PIPES), N-[2-Hydroxyethyl]piperazine-N′-[2-ethanesulfonic acid] (HEPES), Ni-NTA Sepharose 6FF (for His-Tag purification), and associated reagents for enzyme purification were procured from Sangon Biotech. Epilactose was sourced from Aladdin. Lactose and sodium chloride were obtained from Sinopharm Chemical Reagent Co., Ltd. Tryptone and yeast extract were acquired from Oxoid. The gene of Acba-CE (National Center for Biotechnology Information: NCBI accession number is RPJ56071.1) was synthesized by Dynegene Technologies and ligated between the *NdeI* and *XhoI* sites of the pET-28a(+) vector. Subsequently, the plasmid was transformed into *Escherichia coli* BL21(DE3) host cells through heat shock at 42°C, facilitating subsequent protein expression.

### 2.2 Expression of Acba-CE

For recombinant protein production, a nutrient-rich culture solution was prepared containing 1% peptone, 1% NaCl, and 0.5% yeast extract, supplemented with 50 μg/mL of the antibiotic kanamycin. The engineered bacterial strains harboring the target enzyme were initially cultured in this medium for 12–16 h at 37°C. Subsequently, 1 mL of the pre-culture was introduced into 100 mL of fresh medium for large-scale cultivation. When the bacterial density, as measured by spectrophotometric analysis at 600 nm, reached 0.6–0.8 absorbance units, protein expression was initiated by adding IPTG to a concentration of 200 μM. The induction process was carried out at a reduced temperature of 16°C for an extended period of 20 h to facilitate proper protein folding.

### 2.3 Purification of Acba-CE

Following cultivation, bacterial cells were harvested by centrifugation at 6,500 × g for 10 min at 4°C. The pellet was washed twice with ice-cold PBS and resuspended in lysis buffer (50 mM phosphate buffer, 200 mM NaCl, 10 mM imidazole [pH 7.5]) using a 50 mL beaker. Cell lysis was performed with a φ10 mm ultrasonic probe under pulsed conditions (5 s ON/9 s OFF per cycle) at 500 W output power for 15 min total duration, with the suspension maintained below 4°C using an ice bath. The lysate was clarified by centrifugation at 9,500 × g for 10 min at 4°C.

Protein isolation was performed using immobilized metal affinity chromatography with Ni-NTA Sepharose 6FF resin (His-Tag specific, Sangon Biotech) through gravity-flow purification according to the manufacturer’s protocol. The resin (5 mL bed volume) was equilibrated with 10 column volumes (CV) of lysis buffer (50 mM phosphate buffer, 200 mM NaCl, 10 mM imidazole, pH 7.5) at a natural flow rate controlled by column height adjustment. After loading the crude enzyme lysate, the column was sequentially washed with 5 CV of low-imidazole buffer (10 mM imidazole in lysis buffer) followed by 5 CV of intermediate-imidazole buffer (35 mM imidazole in lysis buffer) to remove nonspecifically bound proteins. His-tagged target proteins were eluted with 3 CV of high-imidazole buffer (250 mM imidazole in lysis buffer), and the eluate was collected in 2 mL microcentrifuge tubes. The collected fractions were immediately subjected to buffer exchange using dialysis against 50 mM PIPES (pH 6.0) at 4°C.

Recombinant cell lysate and purified protein was analyzed by 12% SDS-PAGE (150 V for 90 min, Bio-Rad Mini-PROTEAN) with Coomassie Blue staining. Quantitative analysis used a BCA assay with BSA standards, measured at 562 nm.

### 2.4 Enzyme assays

The enzymatic activity of Acba-CE toward lactose was assessed by monitoring substrate conversion through high performance liquid chromatography (HPLC). Reactions were conducted in 0.4 mL systems containing 200 mM lactose dissolved in 50 mM PIPES buffer (pH 6.0, adjusted with 5 M NaOH) with 0.02 mg/mL enzyme. After incubation at 60°C for 15 min in a temperature-controlled thermal cycler, reactions were terminated by boiling at 100°C for 3 min. Terminated samples were centrifuged at 7,000 × g for 3 min. To remove residual proteins, trichloroacetic acid (TCA) was added to a final concentration of 15% (w/v). The clarified supernatants were filtered through 0.22 μm hydrophilic nylon membranes prior to HPLC analysis as described in subsequent sections.

The enzymatic activity of Acba-CE was determined by quantifying epilactose production via HPLC. Enzyme activity units (U) were defined as the amount of enzyme required to generate 1 μmol of epilactose per minute under standard conditions (60°C, pH 6.0), calculated based on epilactose concentrations derived from a 0.1–20 mM calibration curve of purified standard. Negative controls with heat-inactivated enzyme (100°C, 10 min pretreatment) demonstrated negligible background conversion (<2%). All assays included triplicate technical replicates, with inter-experimental variation controlled to <5% coefficient of variation through standardized protocols.

### 2.5 Determination of Acba-CE enzymatic properties

The optimal temperature and pH of Acba-CE were determined by quantifying epilactose production via HPLC across temperature (4–90°C) and pH gradients. Temperature profiling was performed using a calibrated thermal block, with 5 min pre-incubation at each temperature. For pH optimization, three buffer systems were employed: 50 mM sodium acetate–acetic acid (pH 4.0–6.0), 50 mM PIPES (pH 6.0–7.5), and 50 mM HEPES (pH 7.5–8.5). All activity values were calculated based on epilactose generation, quantified against a 0.1–20 mM epilactose calibration curve.

Thermal stability was evaluated by pre-incubating the enzyme at 50–70°C for a period of time in assay buffer. Residual activity was defined as the percentage of epilactose produced relative to untreated controls (100% activity), measured under standard conditions (60°C, pH 6.0).

Michaelis–Menten kinetics were analyzed through nonlinear regression (Origin 2021) using epilactose production rates. Reactions containing 0.02 mg/mL purified enzyme were conducted at pH 6.0 and 60°C with lactose concentrations spanning 30–800 mM (8 gradient points).

### 2.6 Production of epilactose by Acba-CE

The time-dependent catalytic conversion of lactose by Acba-CE was evaluated through parallel reaction systems. For lactose solution studies, individual 1.5 mL microcentrifuge tubes containing 0.4 mL reaction mixtures (0.5 mg/mL enzyme, 200 mM lactose in 50 mM PIPES pH 6.0) were incubated at 50°C. Triplicate samples were independently prepared for each time point (2, 6, 12, 24, 36, and 48 h). At designated intervals, entire reaction volumes (0.4 mL) were heat-inactivated (100°C, 3 min), followed by centrifugation (8,000 × g, 3 min), TCA precipitation, and filtration as per standard protocols.

Milk-based reactions were conducted in 5 mL microcentrifuge tubes with 1 mL systems, comprising 850 μL pasteurized milk (50 g/L carbohydrates) and 150 μL enzyme solution (final 1 mg/mL Acba-CE). Triplicate tubes were incubated at 10°C for 12 h. Reactions were terminated by heating (100°C, 3 min), followed by centrifugation (8,000 × g, 10 min), TCA precipitation, and filtration as per standard protocols. Lactose concentrations were corrected for initial 15% dilution during data normalization.

### 2.7 High performance liquid chromatography (HPLC) analysis

Quantitative analysis of epilactose, lactulose, and lactose was performed using an HPLC system (Waters Alliance e2695, USA) equipped with a differential refractive index detector (RID, Waters 2,414, USA), following chromatographic conditions from previously reported methodologies (Chen et al., 2016). Separation was achieved on a Shodex VG50-4E carbohydrate column (4.6 mm × 250 mm, 5 μm particle size) maintained at 40°C. The mobile phase consisted of acetonitrile/methanol/water (75:20:5, v/v/v) delivered isocratically at 1.0 mL/min. Sample injections of 20 μL were performed using an autosampler after filtration through 0.22 μm nylon membranes. Analyte identification was confirmed by retention time alignment with authentic standards, and quantification utilized external calibration curves.

### 2.8 Phylogenetic analysis

Collect currently reported 19 kinds of CE sequence using the basic local alignment search tool^1^ of Acba-CE and the CE sequence similarity comparison, and use the ClustalW neighborhood connection method to construct phylogenetic tree.

## 3 Results and discussion

### 3.1 Amino acid sequence analysis

Multiple sequence alignments and phylogenetic trees were used to analyze the Acba-CE sequences mined from the UniProtKB TrEMBL database. The Acba-CE sequence was identified through a targeted screening strategy using the UniProtKB query system: initial retrieval of all unreviewed entries annotated as “cellobiose 2-epimerase” (2,265 candidates) followed by taxonomy filtering for *Acidobacteriota* phylum organisms inhabiting acidic environments (5 candidates). The final prioritization is based on structural homology with characterized CE enzymes, preferentially selecting candidates that exhibit higher homology to CE enzymes with lower optimal temperatures and lower optimal pH.

**Figure 2:**
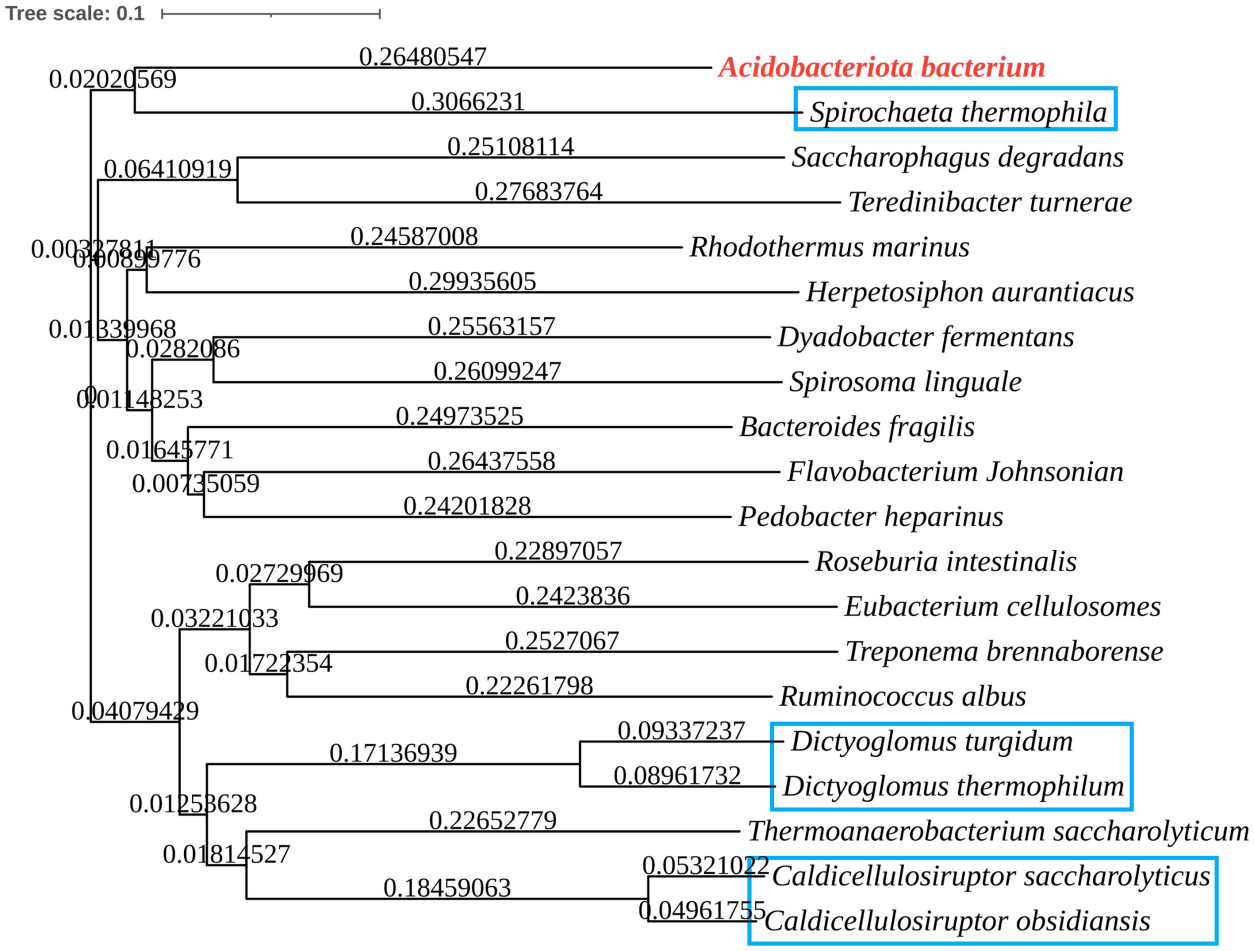
Phylogenetic tree of CEs from various microorganisms, comparing some reported CE enzymes with Acba-CE (blue box for CE producing lactulose).

**Figure 3:**
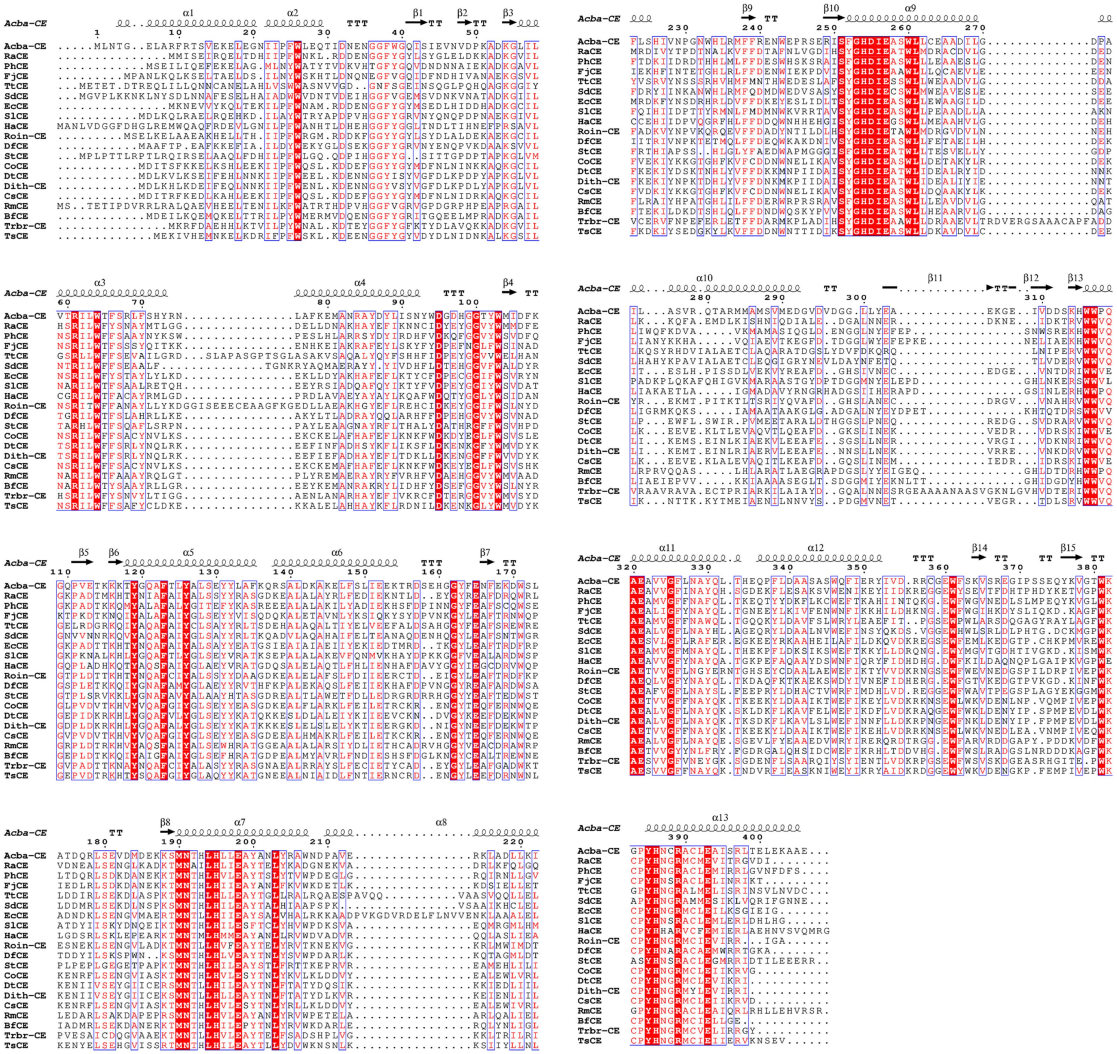
Multiple sequence alignment of the selected CEs. The alignment and its secondary structure analysis were performed using ClustalOmega and Espript 3.0. Three histidines involved in each catalytic center of CEs were symbolized according to the crystal structure of Acba-CE. The strict conserved residues are demonstrated by white characters with red boxes, red characters denote the residues with high similarity in each group. The residues with high similarity across the groups are boxed in blue frames.

The phylogenetic tree in Figure 2 shows that the Acba-CE clusters within a clade containing experimentally validated epimerases, despite moderate global sequence similarity (35–50% identity range). Among the compared CEs, Acba-CE exhibits the highest sequence similarity (46.61%) with the CE enzyme derived from *Pedobacter heparinus* (Krewinkel et al., 2014). According to previously reported literature, PhCE is a mesophilic enzyme with an optimal pH in the mildly acidic range, showing properties closely aligned with the target enzyme in this study (adapted to mildly acidic and low-temperature catalytic environments). Key catalytic residues were fully conserved across aligned sequences (Figure 3), while variable loop regions (e.g., Acba-CE_loop 175–186 and loop 236–248) exhibited less than 20% similarity, potentially explaining substrate specificity differences. Quantitative similarity metrics across functional domains are presented in Table 2.

**Table 2:** Comparison of sequence similarity between Acba-CE and other CE.

Organism	Identities	Length	GenBank	References
*Pedobacter heparinus*	46.61%	397	WP_015809782.1	Krewinkel et al. (2014)
*Rhodothermus marinus*	45.57%	412	ACK49317.1	Ojima et al. (2014)
*Spirochaeta thermophila*	43.43%	406	WP_013312912.1	Park et al. (2013)
*Herpetosiphon aurantiacus*	42.42%	416	WP_012188443.1	Krewinkel et al. (2014)
*Saccharophagus degradans*	41.88%	410	WP_011466993.1	Krewinkel et al. (2014)
*Bacteroides fragilis*	41.65%	392	BAH23773.1	Senoura et al. (2014)
*Thermoanaerobacterium saccharolyticum*	41.54%	392	YP006392930.1	Chen et al. (2015)
*Dyadobacter fermentans*	41.11%	392	WP_015811450.1	Krewinkel et al. (2014)
*Dictyoglomus turgidum*	40.86%	389	ACK41937	Kim and Oh (2012)
*Caldicellulosiruptor saccharolyticus*	40.86%	390	WP_011915904.1	Chen et al. (2016)
*Flavobacterium johnsonian*	40.66%	396	WP_012026921.1	Krewinkel et al. (2014)
*Dictyoglomus thermophilum*	40.36%	389	ACK41937	Ito et al. (2008)
*Spirosoma linguale*	40.15%	402	WP_012924887.1	Krewinkel et al. (2014)
*Caldicellulosiruptor obsidiansis*	40.10%	390	CP002164.1	Xiao et al. (2019)
*Ruminococcus albus*	39.90%	389	ADU20582.1	Kim et al. (2012) and Ito et al. (2007)
*Teredinibacter turnerae*	36.78%	425	WP_015818822.1	Krewinkel et al. (2014)
*Eubacterium cellulosomes*	36.30%	405	EIM57035.1	Taguchi et al. (2008)
*Treponema brennaborense*	36.12%	418	NC_015500.1	Chen et al. (2021)
*Roseburia intestinalis*	35.52%	409	NZ_LR027880.1	Chen et al. (2020)

### 3.2 Expression and purification of Acba-CE

**Figure 4:**
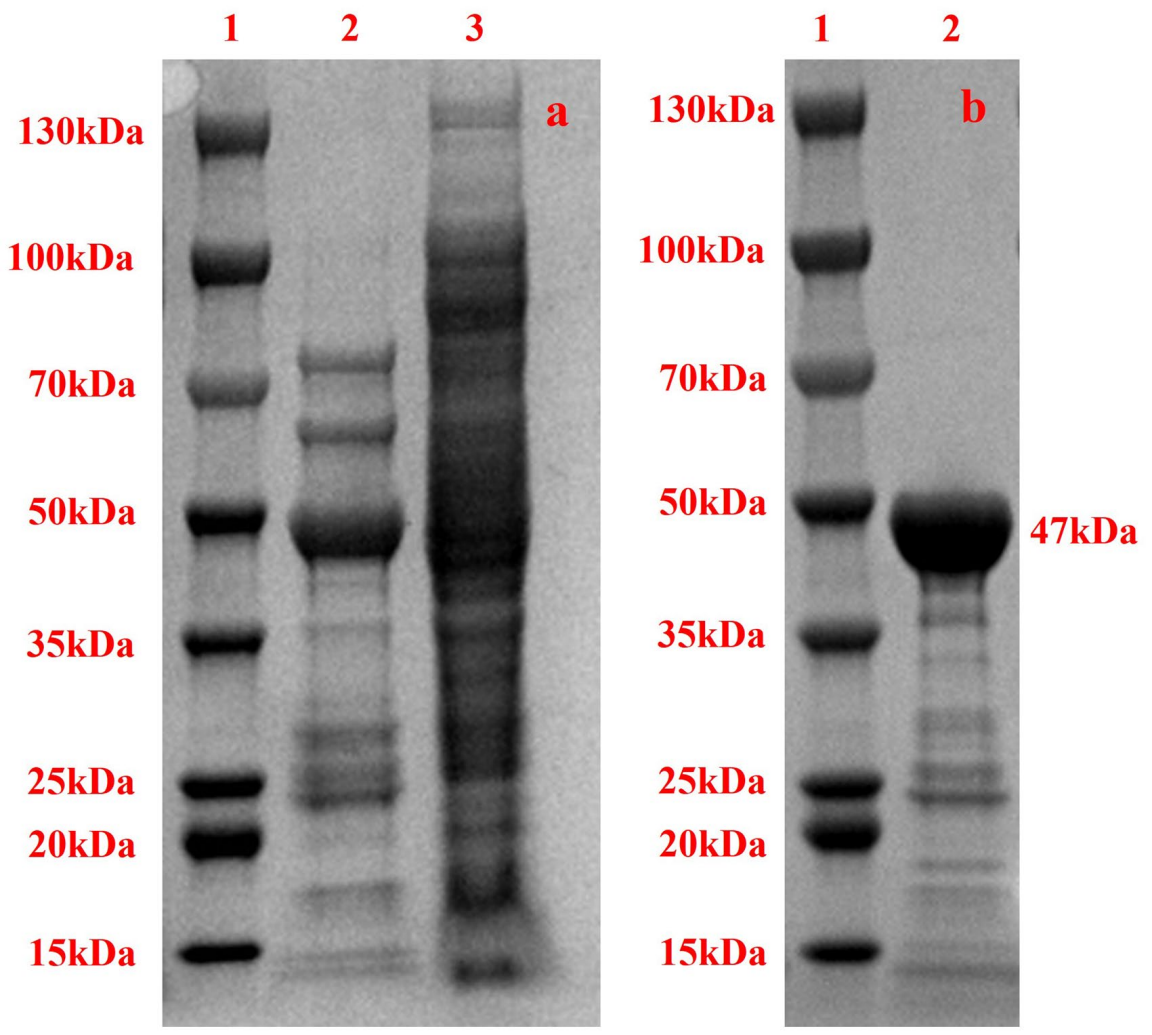
SDS-PAGE of Acba-CE. **(a)** SDS-PAGE analysis of recombinant cell lysate. Lane 1: Protein molecular weight marker. Lane 2: Pellet fraction after cell lysis. Lane 3: Supernatant fraction after centrifugation. **(b)** Recombinant Acba-CE, purified from *E. coli* transformant, were analyzed on SDS-PAGE. Lane 1 indicates molecular mass standard, with the size of each marker indicated in the figure. Lane 2 indicates purified Acba-CE protein.

To validate the expression and solubility of the recombinant protein, we have conducted SDS-PAGE analyses of both the supernatant and pellet fractions of the lysed recombinant cells (as shown in Figure 4a). The electrophoretic results clearly demonstrate successful protein expression, with distinct bands corresponding to the expected molecular weight of the target protein. This experiment confirms the effective expression and partial solubility of the recombinant enzyme, providing critical support for the subsequent functional characterization described in the study.

To obtain pure Acba-CE enzyme for subsequent assays, the recombinant protein with a His tag, induced by IPTG, was purified using a Ni^2+^ column. SDS-PAGE analysis of the purified protein (Figure 4b) revealed that the size of the purified protein was approximately 47 kDa, which is generally consistent with the theoretical size of 47 kDa. Additionally, the electrophoresis diagram demonstrated that the purified solution contained only a small amount of other impurity proteins, making it suitable for further research.

### 3.3 Enzymatic properties of Acba-CE

**Figure 5:**
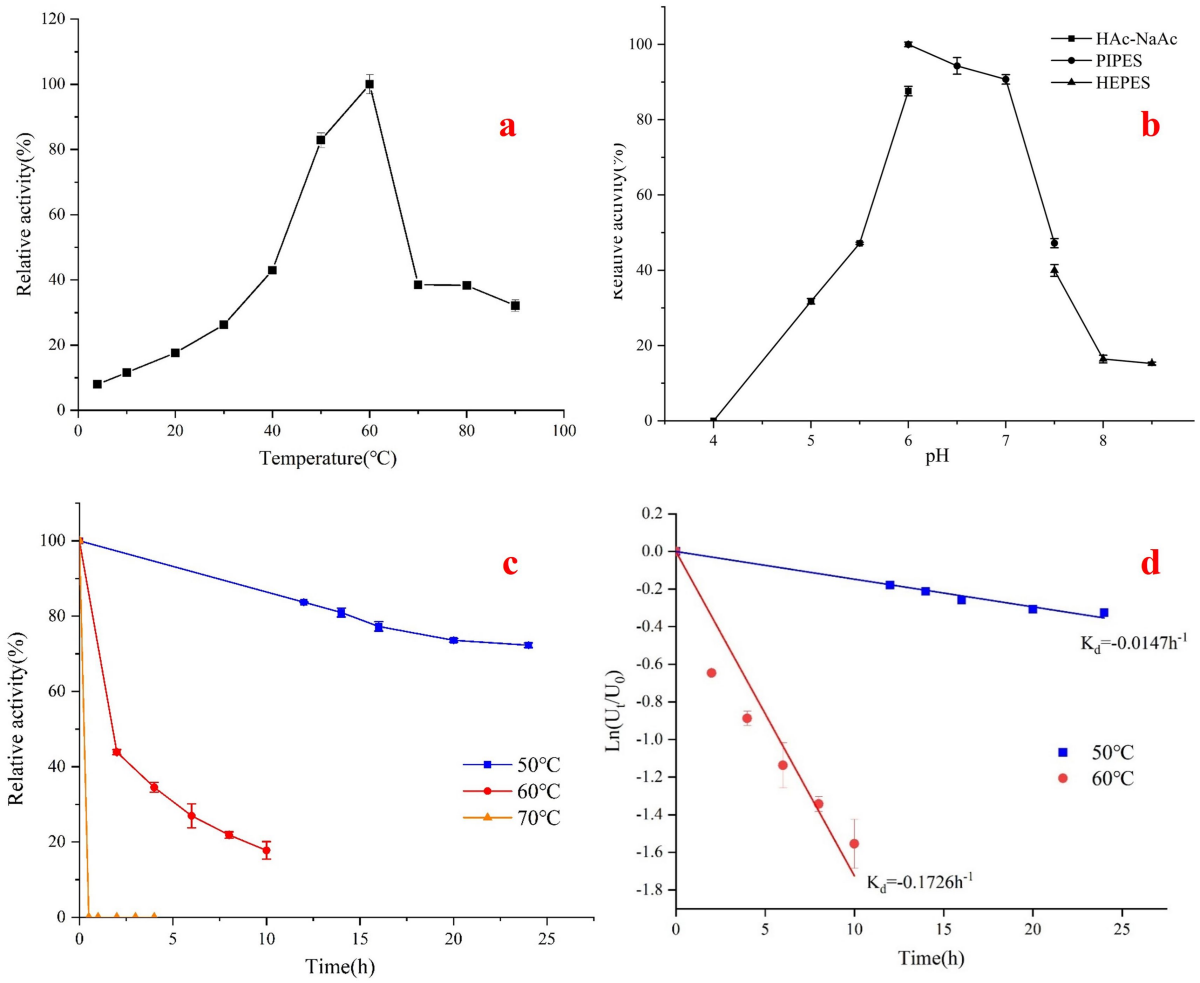
The optimum temperature, pH and thermal stability of Acba-CE were determined. **(a)** Optimal temperature. The reaction was carried out in 50 mM PIPES buffer (pH 6.0) at 4–90°C, supplemented with lactose substrate at a final concentration of 200 mM. **(b)** Optimal pH. The reaction was carried out in 50 mM PIPES buffer (pH 4.0–8.5) at 60°C with a final concentration of 200 mM lactose substrate. **(c)** Thermal stability and **(d)** half-life assays were performed in PIPES buffer (pH 6.0) at 50 mM after treatment at temperatures 50 and 60°C, followed by addition of lactose substrate at a final concentration of 200 mM at 60°C.

The Acba-CE produces epilactose when catalyzing lactose and does not form lactulose, demonstrating the typical characteristics of a mesophilic CE enzyme. The activity of catalyzing lactose to epilactose is 37.3 ± 1.3 U/mg. Under standard assay conditions, the optimal temperature for Acba-CE was found to be 60°C (Figure 5a), which aligns with the general characteristics of mesophilic CE enzymes. While the enzyme exhibits residual activity at 70°C-90°C(~30–40%), its significant activity loss after prolonged incubation indicates limited thermal stability. Additionally, Acba-CE exhibits stronger cold adaptability compared to heat adaptability, with a lesser decrease in enzyme activity when the temperature drops than when it rises, as also reflected in the thermostability assay of Acba-CE (Figures 5c,d). When Acba-CE is incubated at different temperatures, the enzyme activity decreases significantly after incubation at 60°C for a period of time, with a more pronounced decrease than at 50°C, and the enzyme is inactivated after incubation at 70°C for half an hour.

Under standard assay conditions, the optimal pH for Acba-CE was determined to be 6.0 (Figure 5b). Additionally, Acba-CE exhibited high activity within the pH range of 6.0–7.0, with relative activity consistently maintained above 90%. Outside this range, both an increase and a decrease in pH resulted in a significant reduction in enzyme activity. As *Acidobacteriota bacterium* is an acidophile, the enzyme isolated from it also demonstrated a certain degree of acidophilia, making it more suitable for application in slightly acidic environments. The stability of Acba-CE under acidic conditions may be related to the distribution of surface acidic amino acids. According to calculations of the solvent-accessible surface area, the proportion of acidic amino acids on the surface of Acba-CE reaches 27%, whereas the proportion on the commonly used mesophilic RaCE surface is only 22%. The elevated density of surface-exposed acidic amino acids potentially enhances the protein’s capacity for proton binding in acidic environments.

The kinetic parameters of Acba-CE were determined within the range of 30 mM to 800 mM, with a Km value of 147.4 ± 10.6 mM and a kcat of 35.02 ± 0.94 s^−1^ when lactose was used as the substrate.

Compared to the CE enzymes from other sources listed in Table 1, only two CEs exhibit lower optimal pH values. Most CEs have optimal pH ranges in the neutral to alkaline region, and their residual activity decreases to varying degrees when the pH drops to mildly acidic conditions. The most significant activity loss is observed in the enzyme from *Ruminococcus albus* (Ito et al., 2007), which retains only 25% activity at pH 6.5. Although the CE from *Rhodothermus marinus* (Ojima et al., 2014) can catalyze reactions under mildly acidic conditions, it is a thermophilic enzyme unsuitable for low-temperature processing environments and difficult to inactivate by heat. While the CE from *Pedobacter heparinus* (Krewinkel et al., 2014) meets the requirements for mildly acidic and low-temperature processing, Acba-CE demonstrates higher k_cat_ and k_cat_/K_m_ values, indicating superior catalytic efficiency.

### 3.4 Acba-CE catalyzes the production of epilactose from lactose

The catalytic efficiency of Acba-CE was systematically evaluated across distinct operational conditions to assess its adaptability and industrial potential. Due to the poor thermal stability of Acba-CE, the protein was easy to precipitate at 60°C for a long time. The conversion rate of Acba-CE catalyzed lactose to epilactose was determined at 50°C. In lactose solution systems (100 mM, pH 6.0) at 50°C, the enzyme exhibited remarkably rapid kinetics, achieving 28% lactose-to-epilactose conversion within the first 2 h—a value approaching the final equilibrium conversion rate of 30.6% observed after 48 h. Subsequent sampling intervals (6, 12, 24, 36, and 48 h) revealed only marginal increases in conversion (29.1% at 6 h; 30.6% at 48 h), indicating that the majority of substrate turnover occurred within the initial reaction phase. This accelerated equilibration likely stems from conformational flexibility in Acba-CE’s substrate-binding loops.

In contrast, when applied to milk systems (50 g/L lactose, pH 6.7) under refrigeration conditions (10°C), Acba-CE demonstrated robust cold adaptability, attaining 28.5 ± 1.5% conversion after 12 h. Despite a significant decrease in temperature, the enzyme still retained a high conversion rate compared to its near-optimal performance at 50°C. Notably, despite a slight decrease in the equilibrium conversion rate between purified lactose (30.6%) and the complex milk matrix (28.5%), the enzyme maintained a high conversion rate, confirming its adaptability to inhibitory milk components (e.g., casein and calcium ions)—a critical advantage for dairy applications.

The temperature-dependent divergence in reaction kinetics underscores Acba-CE’s operational versatility. While elevated temperatures (50°C) enable rapid substrate turnover for time-sensitive processes, the enzyme’s sustained activity at 10°C aligns with industrial cold-chain requirements. This dual functionality bridges the gap between catalytic efficiency and practical feasibility, allowing seamless integration into both batch processing (e.g., rapid enzymatic modification prior to pasteurization) and prolonged cold storage workflows. The enzyme’s rapid thermal inactivation at 70°C further ensures compatibility with existing dairy sterilization protocols, eliminating the need for post-reaction purification—a persistent challenge for thermostable epimerases.

## 4 Conclusion

This study establishes Acba-CE as a novel cellobiose 2-epimerase with unique functional attributes used for dairy biotechnology applications. By mining the UniProtKB TrEMBL database and prioritizing *Acidobacteriota*-derived sequences, we identified a CE enzyme exhibiting dual advantages of acidophilic activity and temperature-responsive inactivation. Heterologous expression in *E. coli* BL21(DE3) yielded a highly pure enzyme (>95%) with robust catalytic efficiency (Km value of 147.4 ± 10.6 mM and kcat of 35.02 ± 0.94 s^−1^).

Structurally, Acba-CE’s elevated surface density of acidic residues (27% vs. 22% in *R. marinus* CE) correlates with enhanced proton retention in acidic environments, while its flexible loop regions (e.g., residues 175–186) likely contribute to cold adaptability. These traits collectively position Acba-CE as a biocatalyst uniquely suited for minimally processed dairy products, where preserving native flavor profiles and nutritional integrity is paramount.

Acba-CE’s optimal pH of 6.0 and sustained activity (>90%) within the pH 6.0–7.0 range align precisely with the natural acidity of milk (pH 6.5–6.8), eliminating the need for pH adjustment during processing. The enzyme’s 29 ± 1.5% lactose-to-epilactose conversion in raw milk at 10°C—demonstrates its potential for energy-efficient cold-processing workflows. Meanwhile, the heat-labile nature of Acba-CE under high-temperature processing ensures minimal post-process enzymatic activity, thereby eliminating the need for downstream purification to remove residual enzymes—a critical bottleneck in current dairy enzyme modification practices.

Looking forward, Acba-CE’s operational stability in milk matrices and compatibility with refrigeration temperatures open avenues for sustainable dairy processing. Future efforts should focus on loop engineering to enhance substrate affinity and immobilization strategies to extend catalytic longevity, ultimately bridging the gap between laboratory-scale efficacy and industrial-scale implementation.

## Data Availability

The datasets presented in this study can be found in online repositories. The names of the repository/repositories and accession number(s) can be found in the article/supplementary material.

## Footnotes

^1^ http://blast.ncbi.nlm.nih.gov/Blast.cgi
